# Comparison of clinical outcomes after drug-eluting stent implantation in diabetic versus nondiabetic patients in China: A retrospective study

**DOI:** 10.1097/MD.0000000000006647

**Published:** 2017-04-28

**Authors:** Yong-Jin Jiang, Wei-Xing Han, Chao Gao, Jun Feng, Zheng-Fei Chen, Jing Zhang, Chun-Miao Luo, Jian-Yuan Pan

**Affiliations:** a Department of Cardiology, the Affiliated Hefei Hospital of Anhui Medical University (The 2nd People's Hospital of Hefei); b Department of Cardiology, the first Affiliated Hospital of Anhui Medical University, Hefei, P.R. China.

**Keywords:** Chinese patients, coronary artery disease, diabetes mellitus, drug-eluting stent, percutaneous coronary intervention

## Abstract

Diabetes mellitus (DM) has been proved to be a predictor of adverse outcomes after percutaneous coronary intervention (PCI). Drug-eluting stents (DESs) could reduce the adverse events in DM patients. In this study, we aimed to analyze the clinical outcome after DES implantation in diabetic versus nondiabetic patients in China. Totally, 200 Chinese DM patients and 400 Chinese non-DM patients were enrolled in this retrospective study. Compared with non-DM patients, DM patients were more likely to have a higher incidence of cardiac death (3.5% vs. 1.0%, *P* = .048), stent thrombosis (2.5% vs. 0.5%, *P* = .044), target lesion revascularization (6.0% vs. 1.8%, *P* = .005), target vessel failure (15.5% vs. 8.0%, *P *< .001), target lesion failure (14.0% vs. 4.3%, *P *< .001), myocardial infarction (4.5% vs. 1.5%, *P* = .030), and major adverse cardiac events (12.5% vs. 5.0%, *P* = .001) at 2-year follow-up. However, the incidence of target vessel revascularization (7.5% vs. 5.5%, *P* = .340) was similar between DB and non-DB patients. Patients with DB (hazard ratio [HR] = 2.54, *P* = .001), older than 80 years (HR = 1.33, *P* = .027) with hypercholesterolemia (HR = 1.03, *P *< .001), serum creatinine >177 μmol/L (HR = 3.04, *P* = .011), a history of cerebral vascular accident (HR = 4.29, *P* = .010), or a history of myocardial infarction (HR = 31.4, *P *< .001) were more likely to experience adverse events. In China, DM could also be served as an independent predictor of adverse outcomes after DES implantation. These patients should be reexamined more frequently.

## 1 Introduction

More than 347 million individuals were affected by diabetes mellitus (DM) globally in the year 2008.^[1]^ In the 1970s, DM was defined as an independent risk factor for cardiovascular disease and mortality.^[2–4]^ High blood glucose levels in DM patients could facilitate and accelerate the atherosclerotic process through different mechanisms.^[5]^ The most common therapy for coronary artery disease patients is percutaneous coronary intervention (PCI).^[6]^

DM has been proved to be a predictor of adverse outcomes after PCI including restenosis, repeat revascularization, myocardial infarction (MI), and mortality.^[7–11]^ Recently, drug-eluting stents (DESs) have been proved to reduce the incidence of restenosis and the need for repeat revascularization when compared with bare-metal stents in patients with DM.^[12,13]^ However, compared with non-DM patients, DM patients still have a high risk of major adverse cardiac events (MACEs) with DES.^[14–16]^ Some studies found that the incidence of cardiac death between patients with or without DM is similar.^[17]^ But some other large sample clinical trials found that patients with DM still had a higher risk of MACE even with DES.^[18–20]^ All studies above are not reported in China.

Thrombotic and thrombolytic status is different between western people and Asians. Compared with patients in western countries, platelets and thrombolysis are both inhibited in Asian patients.^[21]^ This may be important in the etiology of thrombotic events. In Chinese patients, coagulation state is not that high, and little data are known about the outcomes of DES usage in Chinese patients.

Thus, we conducted this study to investigate the efficiency and safety of DES in Chinese patients with or without DM. Furthermore, we also conducted a meta-analysis to systematically review the effect of DM in patients undergoing PCI with DES.

## 2 Patients and methods

### 2.1 Ethics statement

This study was approved by the institutional review board of Anhui Medical University, and it was conducted in accordance with the Declaration of Helsinki and internationally accepted ethical guidelines. All patients signed an informed consent form.

### 2.2 Patients

Totally, 600 Chinese patients treated with PCI and with DES were continuously enrolled in this retrospective study between 2010 and 2013 (DM, n = 200; non-DM, n = 400). Patients included in this study should meet the following criteria: age 18 to 80 years; clinically diagnosed as having chronic coronary artery disease (CAD) or acute coronary syndrome (ACS); >1 target lesion; target lesion stenosis >50%; using at least 1 DES. Exclusion criteria were: history of stroke or transient ischemic attack within 6 months; history of coagulopathy; platelet count <100,000 or >700,000 cells/mm^3^ or a WBC count <3000 cells/mm^3^.

For patients with stable CAD, those experiencing the following indications were eligible for PCI: target lesion stenosis ≥70%; target lesion stenosis is <90% but fractional flow reserve <0.8. For patients with non–ST-segment elevation ACS, patients experiencing the following indications were eligible for PCI: unstable hemodynamics patients; refractory angina pectoris; patients with life-threatening arrhythmia; acute heart failure; with increased serum troponin levels. PCI was conducted as soon as possible when patients experienced ST-segment elevation myocardial infarction.

### 2.3 PCI and periprocedure management

The following information was collected for patients included in our study: age, sex, height, weight, blood pressure, blood glucose level, blood lipid levels, serum creatinine levels, electrocardiography, and disease history (history of hypertension, hypercholesterolemia, smoking, myocardial infarction, PCI, cerebral vascular accident, and family history of coronary disease).

PCI was performed according to current standard procedural guidelines.^[22]^ Quantitative coronary angiography was performed using the Philips quantitative coronary analysis system (Toshiba, Japan) and was assessed by an interventional cardiologist. A successful procedure was defined as <25% residual stenosis after PCI.

Before or during the PCI procedure, all patients received at least 300 mg of aspirin and a 300- to 600-mg loading dose of clopidogrel. After the PCI procedure, all patients were given 100 mg/day of aspirin continuously and 75 mg/day clopidogrel for at least 12 months.

### 2.4 DM

DM was diagnosed as an abnormal blood glucose level (=126�mg/dL) after an overnight fast, an abnormal glycosylated hemoglobin test (=6.5%), or an abnormal glucose-tolerance test (2 hours =200�mg/dL).^[23]^ DM patients were further stratified by DM treatment into insulin-treated DM (ITDM) and noninsulin-treated DM (NITDM). For NITDM patients, treatments were oral glucose-lowering drugs or without medication, such as lifestyle modification.

### 2.5 Outcomes and follow-up

The following information was collected at 2-year follow-up: death (cardiac death and death of other reasons), stent thrombus (at 1 month/1 year/2 year), target vessel failure (TVF), target lesion failure (TLF), target vessel revascularization (TVR), target lesion revascularization (TLR), MI, and MACE. Endpoint events were defined as death. Stent thrombus were assessed according to the Academic Research Consortium definition.^[24]^ TLF is composed of cardiac death, target vessel-related MI, and TLR. And TVF is composed of cardiac death, target vessel-related MI, and TVR. MACEs were defined as cardiac death plus stent thrombus plus TVR.

### 2.6 Statistical analysis

SPSS 20.0 (IBM, Chicago, IL) was used for statistical analysis and *P* value <.05 was defined as the threshold of statistical significance. For normally distributed data, mean ± standard deviation (SD) was used for statistics. And asymmetrically distributed data were expressed as median (range). Independent-sample *t* tests were used to calculate the differences between the 2 therapies. The Kaplan-Meier method was used to evaluate the incidence of cumulated events. Univariable and multivariable Cox proportional hazard regression models were used to compare time-dependent dichotomous events among groups.

### 2.7 Systematic review and meta-analysis

MEDLINE, EMBASE, Science Citation Index, the Cochrane Library were systematically searched using the following Keywords: drug eluting stent or DES; diabetes mellitus or DM; percutaneous coronary intervention or PCI.

RevMan 5.2.6 (Cochrane Collaboration) were used for meta-analysis. Mantel-Haenszel risk ratios (RRs) with corresponding 95% confidence intervals (CIs) were calculated for the pooled outcomes. Heterogeneity was assessed by calculating *I*^*2*^. Homogeneity between trials was assessed using the *χ*^2^ test with the significance threshold set at *P *> .1.

## 3 Results

### 3.1 Characteristics of the study population

Totally, 600 patients including 200 DM (ITDM, n = 68; NITDM, n = 132) and 400 non-DM patients treated with PCI were enrolled in our study. Compared with non-DM patients, DM patients had higher body mass index (BMI), and were more likely to have a history of hypertension, hypercholesterolemia, peripheral vascular disease, kidney function deficiency (serum creatinine >177 μmol/L), and family history of coronary disease. In DM patients, all baseline characters were similar between ITDM and NITDM, except that ITDM patients were more likely to have kidney function deficiency (serum creatinine >177 μmol/L) (Table 1).

**Table 1:**
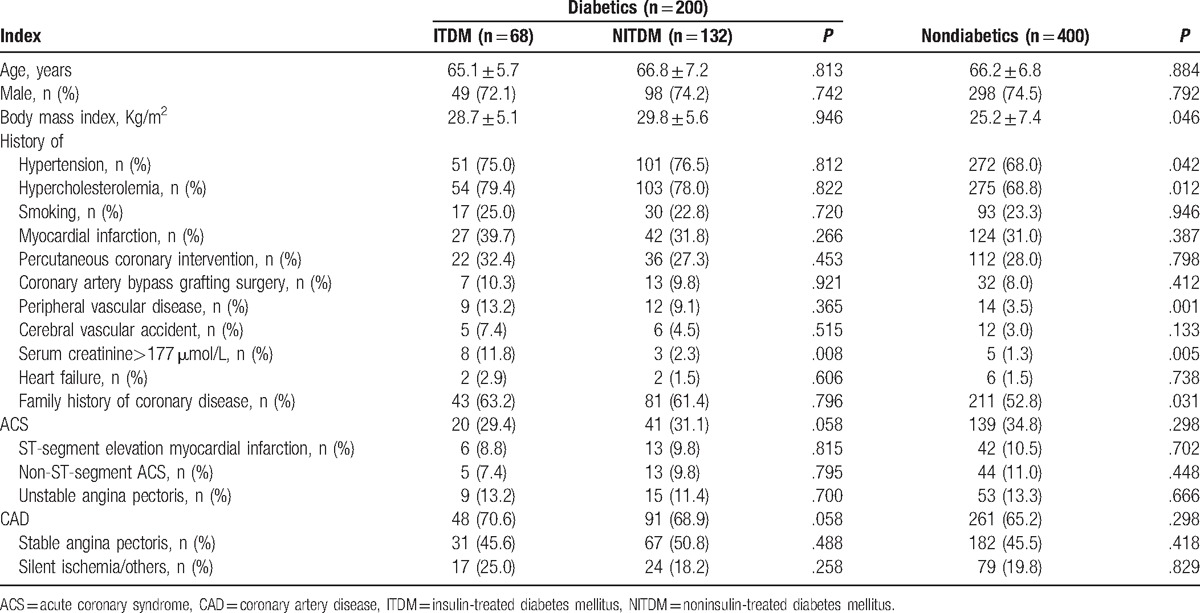
Baseline characteristics of included patients.

### 3.2 Angiographic and procedural characteristics

In angiographic findings, non-DM patients were more likely to have less 1-vessel disease and 3-vessel disease. Compared with DM patients, the stents’ number and length were significantly smaller and shorter for non-DM patients and maximal pressure was lower in non-DM patients. The differences of angiographic findings, lesion classification, and stent characteristics were similar between ITDM and NITDM patients (Table 2).

**Table 2:**
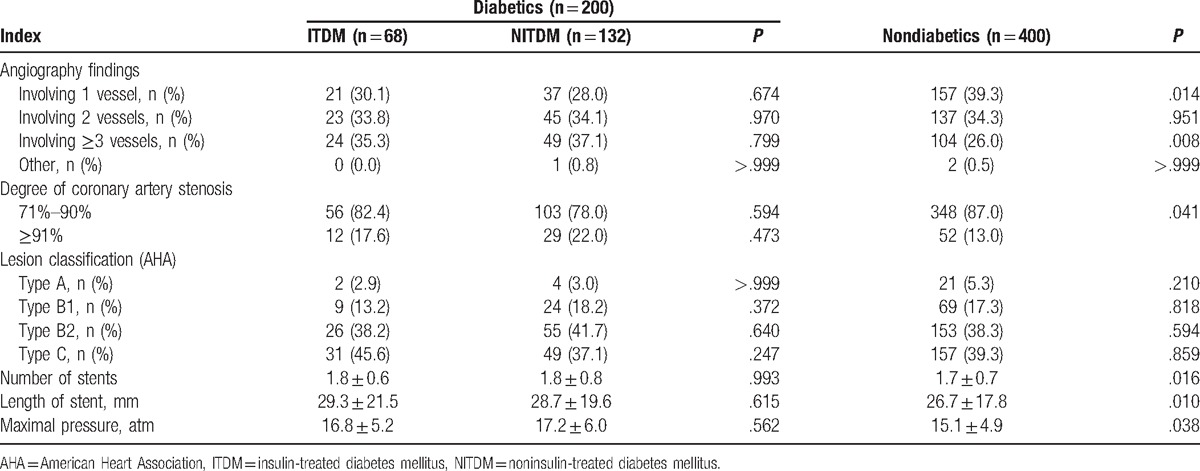
Angiographic and procedural characteristics of enrolled patients.

### 3.3 Outcomes at 2 years’ follow-up

#### 3.3.1 Death

**Figure 1:**
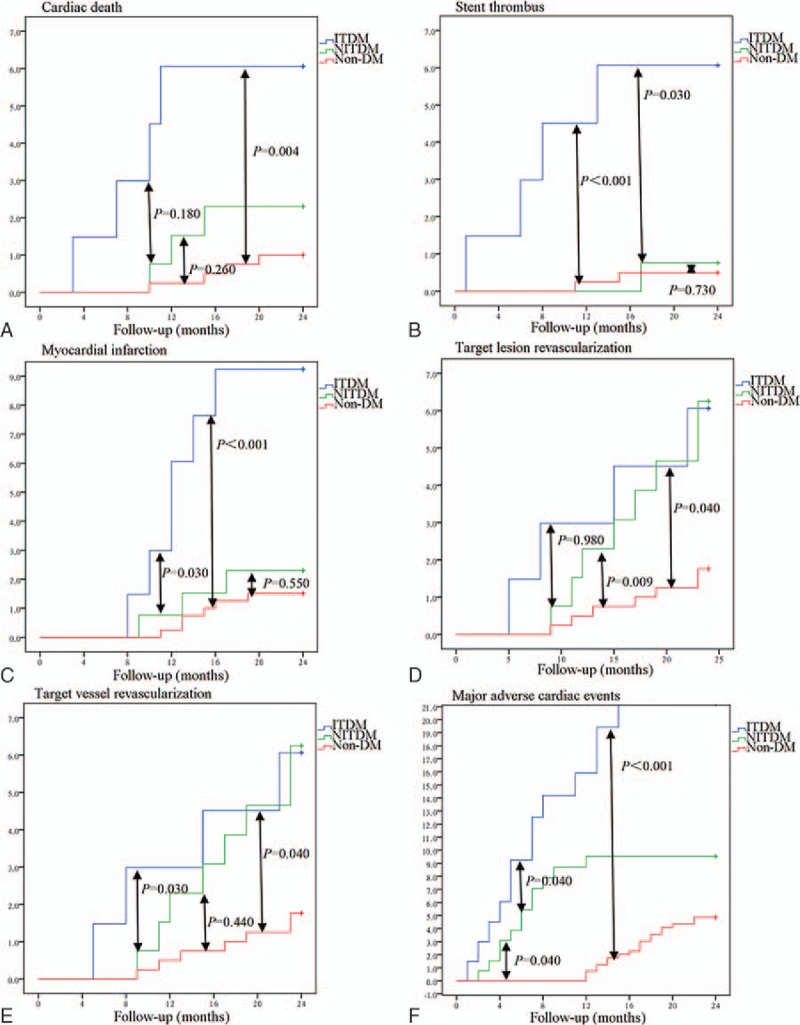
Cumulative incidence of outcomes at 2-year follow-up.

All patients were eligible for the information collection at 2 year's follow-up. Totally 19 deaths were detected (ITDM, n = 5; NITDM, n = 5; non-DM, n = 9). Together, 11 patients (ITDM, n = 4; NITDM, n = 3; non-DM, n = 4) experienced cardiac death. The incidence of cardiac death was significantly lower in non-DM patients group (1.0%) compared with DM patients (3.5%). But the difference was similar between ITDM (5.9%) and NITDM patients (2.2%) (Table 3). Cumulative incidence of cardiac death was significantly higher in ITDM group compared with non-DM patients (*P* = .004), and the difference between ITDM and NITDM patients group (*P* = .180) or between NITDM and non-DM patients group (*P* = .260) was not significant (Fig. 1).

**Table 3:**
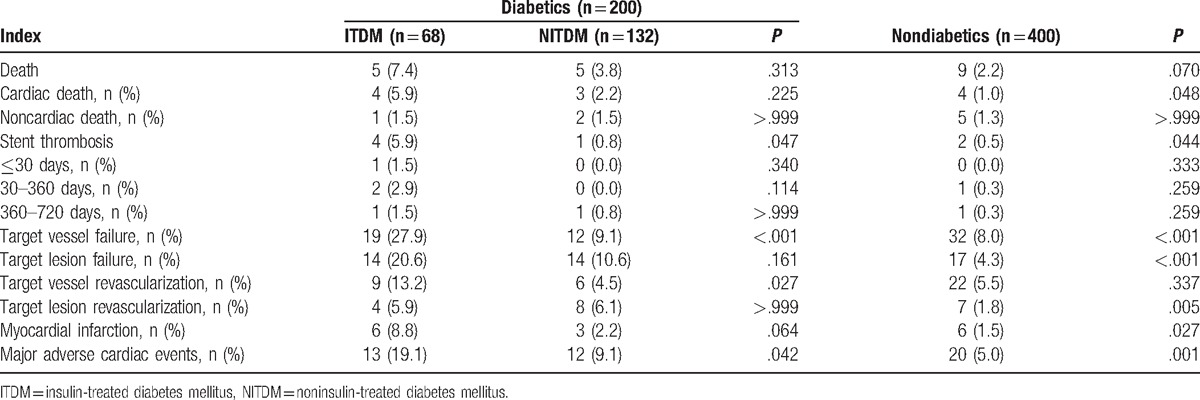
Adverse events at 2-year follow-up.

#### 3.3.2 Stent thrombosis

Totally, 5 DM patients (ITDM, n = 4; NITDM, n = 1) and 2 non-DM patients were found to have stent thrombus (*P* = .040). The difference between DM and non-DM patients or ITDM and NITDM patients at follow-up of 1 month, 1 month to 1 year, and 1 year to 2 year was of no significance (Table 3). Cumulative incidence of stent thrombus was significantly higher in ITDM patients group when compared with non-DM patients (*P *< .001) or NITDM patients (*P* = .030). However, the difference between NITDM and non-DM patients group (*P* = .730) was not significant (Fig. 1).

#### 3.3.3 TVF/TLF

Compared with DM patients (ITDM, n = 19; NITDM, n = 12), non-DM patients (n = 32) experienced significantly less incidence of TVF (*P *< .001). Nevertheless, non-DM patients (n = 17) also experienced significantly less incidence of TLF compared with DM patients (ITDM, n = 14; NITDM, n = 14) (*P *< .001) (Table 3).

#### 3.3.4 TVR/TLR

TVR was detected in a total of 15 DM patients (ITDM, n = 9; NITDM, n = 6) and 22 non-DM patients (*P* = .340). Compared with non-DM patients (n = 7), more DM patients (ITDM, n = 4; NITDM, n = 8) experienced TLR (*P* = .005). Both the differences of TVR and TLR between ITDM and NITDM patients were not significant (Table 3).

Cumulative incidence of TLR was significantly lower in non-DM group when compared with ITDM group (*P* = .040) or NITDM patients group (*P* = .009). The difference between ITDM and NITDM group (*P* = .980) was not significant. Cumulative incidence of TVR was significantly higher in ITDM patients when compared with non-DM patients (*P* = .040) or NITDM patients (*P* = .030). However, the difference between NITDM and non-DM patients (*P* = .440) was not significant (Fig. 1).

#### 3.3.5 Myocardial infarction

The incidence of MI was significantly lower in non-DM patients (n = 6) than DM patients (ITDM, n = 6; NITDM, n = 3) (*P* = .03). The difference between ITDM and NITDM patients was not significant (Table 3). Cumulative event incidence of MI was significantly higher in ITDM patients when compared with non-DM patients (*P *< .001) or NITDM patients (*P* = .030). However, the difference between NITDM and non-DM group (*P* = .550) was not significant (Fig. 1).

#### 3.3.6 MACEs

Non-DM (n = 20) patients experienced significantly lower incidence of MACE than DM patients (ITDM, n = 13; NITDM, n = 12) (*P* = .001). Moreover, NITDM patients experienced lower incidence of MACE than ITDM patients (*P* = .040) (Table 3). Cumulative event incidence of MACE was significantly higher in ITDM patients group when compared with non-DM patients (*P *< .001) or NITDM patients (*P* = .040). Moreover, the difference between NITDM and non-DM group (*P* = .040) was also significant (Fig. 1).

#### 3.3.7 Risk factors for cardiac death/stent thrombosis/MACEs

Patients with DM (hazard ratios [HRs] = 2.54, 95% CI 1.429–4.514, *P* = .001], older than 80 years (HR = 1.33, 95% CI 0.125–1.885, *P* = .027), with hypercholesterolemia (HR = 1.03, 95% CI 1.017–2.066, *P *< .001), serum creatinine >177 μmol/L (HR = 3.04, 95% CI 1.291–7.180, *P* = .011), a history of cerebral vascular accident (HR = 4.29, 95% CI 1.422–12.967, *P* = .010), or with a history of MI (HR = 31.4, 95% CI 11.429–86.273, *P *< .001) were more likely to experience adverse events (Table 4).

**Table 4:**
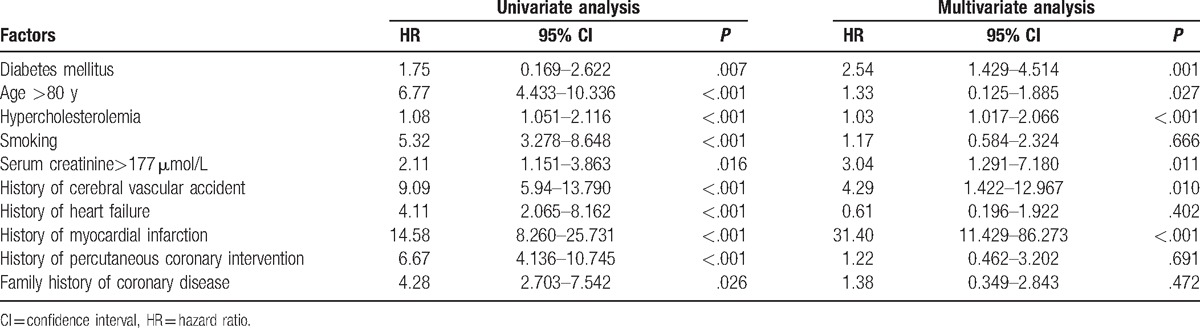
Risk factors for death/ major adverse cardiac events/stent thrombosis in patients with or without diabetes mellitus.

### 3.4 Systematic review and meta-analysis

After systematically reviewing the following database (MEDLINE, EMBASE, Science Citation Index, and the Cochrane Library), the authors found that 22 studies reported the effect of DES both in DM and non-DM patients.^[7–11,14–20,25–34]^ However, in Fröbert et al's study,^[30]^ detailed data about adverse events were not reported. Patients’ data of Kereiakes et al's study^[32]^ were extracted from SPIRIT IV Clinical Trial which was part of Stone et al's study.^[33]^ Moreover, we also found that patients’ data of Sato et al's study^[31]^ was part of their latter study.^[27]^ Thus, we excluded the 3 studies above, and conducted a meta-analysis with the other 19 studies^[7–11,14–20,25–29,33,34]^ and our own data.

#### 3.4.1 Cardiac Death

**Figure 2:**
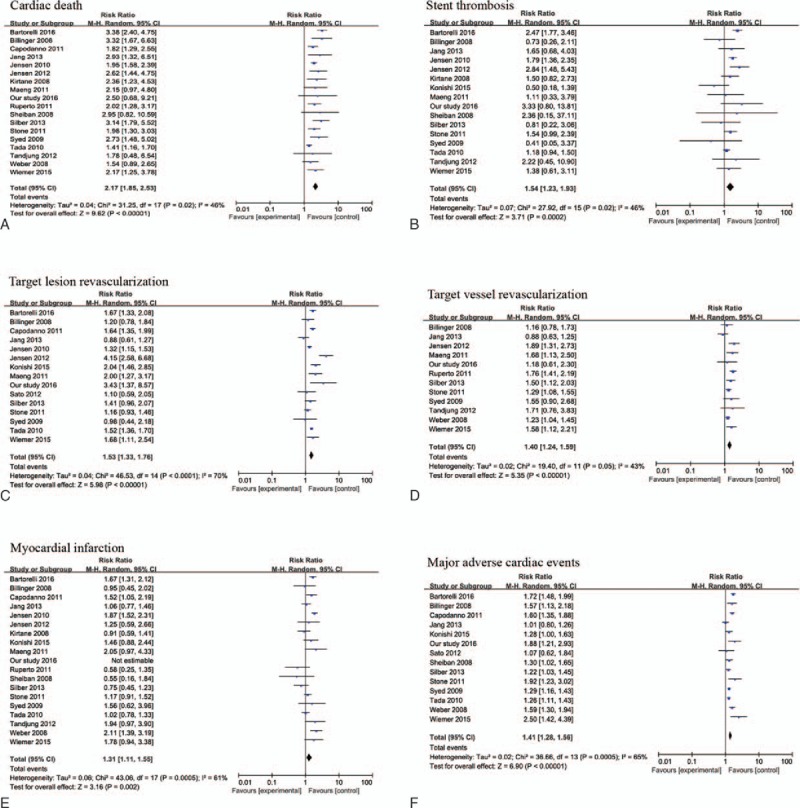
Meta-analysis of included studies.

A total of 17 studies^[7–9,11,14–20,25,26,28,29,33,34]^ and the data in the present study reported cardiac death; the pooled analysis showed that DM patients had a higher incidence of cardiac death (RR = 2.17, 95%CI 1.85–2.53, *I*^*2*^ = 46%) (Fig. 2).

#### 3.4.2 Stent thrombosis

Totally 15 studies^[7–11,14,15,17–19,26,28,29,33,34]^ and our data reported stent thrombus; the pooled analysis showed that DM patients had a higher incidence of stent thrombus (RR = 1.54, 95% CI 1.23–1.93, *I*^*2*^ = 46%) (Fig. 2).

#### 3.4.3 TVR/TLR

Totally 15 studies^[7–11,14,18,25–29,33,34]^ and our data reported TLR; the pooled analysis showed that DM patients had a higher incidence of TLR (RR = 1.53, 95% CI 1.33–1.76, *I*^*2*^ = 70%). Eleven studies^[7,8,11,14,16–18,20,26,28,33]^ as well as our data reported TVR; the pooled analysis showed that DM patients had a higher incidence of TVR (RR = 1.40, 95% CI 1.24–1.59, *I*^*2*^ = 43%) (Fig. 2).

#### 3.4.4 MI

Totally 18 studies^[7–11,14–20,25,26,28,29,33,34]^ and our data reported MI; the pooled analysis showed that DM patients had a higher incidence of MI (RR = 1.31, 95% CI 1.11–1.55, *I*^*2*^ = 61%) (Fig. 2).

#### 3.4.5 MACEs

Totally 13 studies^[7–11,14,19,20,25,27–29,33]^ and the investigation herein reported MACE; the pooled analysis showed that DM patients had a higher incidence of MACE (RR = 1.41, 95% CI 1.28–1.56, *I*^*2*^ = 65%) (Fig. 2).

## 4 Discussion

Compared with non-DM patients, DM patients tend to have lesions in small vessels,^[35]^ which remains an important predictor of restenosis even in DES.^[36]^ Therefore, in patients treated with PCI using DES, DM is still an independent risk factor for worse outcomes. In our study, we found that DM patients were more likely to have poor prognostic outcomes and higher incidence of adverse events.

### 4.1 DM patients versus non-DM patients

Compared with bare metal stents, DES has been proved to reduce neointimal hyperplasia and therefore restenosis rates in DM patients.^[8,37,38]^ However, patients with DM continue to be an independent risk subset associated with worse clinical outcomes.^[39]^

In our study, we found that patients with DM experienced more cardiac death (3.5% vs. 1.0%). Our result was in accordance with other studies (cardiac death rate varied from 1.1% to 12.4% in DM patients, 0.6% to 4.6% in non-DM patients).^[7–9,11,14–20,25,26,28,29,33,34]^ Syed et al^[28]^ showed a relatively high death rate (DM patients: 12.4%; non-DM patients: 4.6%). This may be attributed to their population characteristics. In their study, we found patients were more likely to have hyperlipidemia (81.3%), more history of chronic renal insufficiency (12.8%) and more history of MI (73.2%), and all these factors were independent risk factors which were associated with cardiac death in our study. Thus, the death rate would be higher in their study.

We also found that a higher incidence of adverse events could be observed in DM patients (stent thrombus, 2.5% vs. 0.5%; TLR, 6.0% vs. 1.8%; MI, 4.5% vs. 1.5%; MACE, 12.5% vs. 5.0%). We also conducted a meta-analysis to figure out the relation between DM and adverse events. From the pooled results, we found that DM patients had a higher incidence of cardiac death, a higher incidence of stent thrombus, a higher incidence of TLR, a higher incidence of TVR, a higher incidence of MI, and a higher incidence of MACE. Based on the results, we believed that DM indeed affected the outcomes after PCI even with DES.

Autopsy and angiographic studies have demonstrated that DM patients were more likely to have higher rates of left main stenosis, chronic total occlusions, diffuse and multivessel disease, smaller vessel size, and longer lesion length.^[40,41]^ All these factors may impact the following revascularization. A greater plaque burden, higher propensity to plaque rupture,^[42]^ enhanced prothrombotic status,^[43]^ exuberant neointimal hyperplasia,^[44]^ more aggressive pattern of atherosclerosis, and endothelial dysfunction^[45]^ could be seen in the inflammatory environments in DM patients. and all these features would help to prompt the occurrence of restenosis after PCI. All results above would explain the reasons why DM patients experienced more adverse events.

### 4.2 ITDM patients versus NITDM patients

At 2-year follow-up, ITDM patients were more likely to experience stent thrombus, TVR, and MACE. Jain et al^[46]^ showed that insulin therapy was not statistically associated with increased propensity for stent thrombus, although ITDM remained at higher risk for other adverse cardiovascular events, which was similar to our study. Baseline characteristics were similar between ITDM and NITDM patients except more ITDM patients experienced renal insufficiency (11.8% vs. 2.3%), and this may explain the increased risk of stent thrombus and other adverse events.

For DM patients, insulin resistance has been associated with detrimental biological processes such as impaired vascular production of nitric oxide and increased levels of endothelin-I and angiotensin-II.^[47]^ Insulin has both proatherogenic and antiatherogenic properties, which would differentially modify the risk of cardiovascular events, depending on the presence of insulin resistance and hyperinsulinemia.^[48]^

In terms of the cumulative risk of adverse events, the risk of NITDM patients and non-DM patients was almost similar (cardiac death, *P* = .263; stent thrombus, *P* = .995; MI, *P* = .548; TVR, *P* = .439; cardiac death, *P* = .263) except TLR (cardiac death, *P* = .009) and MACE (cardiac death, *P* = .049). The observation that the risk of serious cardiovascular events was similar between NITDM and non-DM patients might have some clinical implications in selecting the coronary revascularization strategy for patients with DM.

We have several limitations in our study. First, the study design is retrospective. In retrospective study, selection bias would occur. But our baseline characteristics were similar between 2 groups. This may reduce the bias to some extent. Second, sample size was not large. Since the data were from a single center, unlike those from multiple-center collaboration, the limitations existed. However, the data reflected our own experience in Chinese population and indeed more future studies with large sample size and pooled results from multiple center were needed to confirm our results.

## 5 Conclusions

DM could be served as an independent predictor of adverse outcomes after drug-DES implantation. These patients should be reexamined more frequently and pay more attention to their stents.

## Acknowledgments

The authors thank Dr. Jonas Cook for language editing.
